# Proprotein Convertase Subtilisin/Kexin Type 9, Brain Cholesterol Homeostasis and Potential Implication for Alzheimer’s Disease

**DOI:** 10.3389/fnagi.2019.00120

**Published:** 2019-05-22

**Authors:** Maria Pia Adorni, Massimiliano Ruscica, Nicola Ferri, Franco Bernini, Francesca Zimetti

**Affiliations:** ^1^Dipartimento di Scienze degli Alimenti e del Farmaco, Università di Parma, Parma, Italy; ^2^Dipartimento di Scienze Farmacologiche e Biomolecolari, Università degli Studi di Milano, Milan, Italy; ^3^Dipartimento di Scienze del Farmaco, Università degli Studi di Padova, Padova, Italy

**Keywords:** PCSK9 (proprotein convertase subtilisin/kexin type 9), Alzheimer, cholesterol, apolipoprotein E, neuron, brain, cognitive, apoE receptors

## Abstract

Alzheimer’s disease (AD) has been associated with dysregulation of brain cholesterol homeostasis. Proprotein convertase subtilisin/kexin type 9 (PCSK9), beyond the known role in the regulation of plasma low-density lipoprotein cholesterol, was first identified in the brain with a potential involvement in brain development and apoptosis. However, its role in the central nervous system (CNS) and in AD pathogenesis is still far from being understood. While *in vitro* and *in vivo* evidence led to controversial results, genetic studies apparently did not find an association between PCSK9 loss of function mutations and AD risk or prevalence. In addition, a potential impairment of cognitive performances by the treatment with the PCSK9 inhibitors, alirocumab and evolocumab, have been excluded, although ongoing studies with longer follow-up will provide further insights. PCSK9 is able to affect the expression of neuronal receptors involved in cholesterol homeostasis and neuroinflammation, and higher PCSK9 concentrations have been found in the cerebrospinal fluid (CSF) of AD patients. In this review article, we critically examined the science of PCSK9 with respect to its modulatory role of the mechanisms underlying the pathogenesis of AD. In addition, based on literature data, we made the hypothesis to consider brain PCSK9 as a negative modulator of brain cholesterol homeostasis and neuroinflammation and a potential pharmacological target for treatment.

## Introduction

The Proprotein convertase subtilisin/kexin type 9 (PCSK9), acts as one of the major regulators of cholesterol homeostasis, by mediating the degradation of hepatic low density lipoprotein receptors (LDLr) (Macchi et al., 2019). Interestingly, PCSK9 was firstly identified in the brain where its expression in primary embryonic telencephalon cells was maximal between embryonic days 13–15, a gestational period characterized by intense neurogenesis (Seidah et al., 2003). In zebrafish, but not in mice, specific knockdown of PCSK9 mRNA led to a general disorganization of cerebellar neurons and loss of hindbrain-midbrain boundaries, with an end result of embryonic death (Poirier et al., 2006).

In this review article, we will focus on the role of PCSK9 at the cerebral level with particular attention on its potential involvement in neuronal functions and Alzheimer’s disease (AD) pathogenesis. We also critically examined the possibility to consider PCSK9 as a modulator of brain cholesterol homeostasis and inflammation and a potential pharmacological target for neurodegenerative disorders.

## Brain Cholesterol Homeostasis

Cholesterol is one of the most important molecules in brain physiology (Chang et al., 2017): it is an important component of myelin, it is involved in neuronal development, synaptogenesis, outgrowth of neuritis, maintenance and repair of damaged membranes (Dietschy, 2009). Due to the presence of the blood-brain barrier (BBB), the brain relies on *in situ* local cholesterol synthesis (Björkhem and Meaney, 2004). In fact, cholesterol cannot cross the BBB, unlike its side-chain oxidized metabolites, 24S-hydroxycholesterol and 27-hydroxycholesterol (Björkhem et al., 2019). Central nervous system (CNS) cells are able to synthesize cholesterol but adult neurons progressively loose this capacity and become dependent on cholesterol provided from astrocytes (Dietschy and Turley, 2004; Saito et al., 2009). Depletion of neuronal cholesterol leads to excess tau phosphorylation, changes in β-amyloid (Aβ) peptides metabolism, neural oxidative stress reactions, ultimately resulting in neurodegeneration, as demonstrated in *ex vivo* rat hippocampus slices (Koudinov and Koudinova, 2005). The transport of cholesterol from astrocytes to neurons is warranted by peculiar molecules and receptors that cooperate in a coordinated manner. Cholesterol produced from astrocytes undergoes cholesterol efflux to Apolipoprotein E (ApoE)-containing particles through the activity of transporters, such as the ATP binding cassette transporters A1 (ABCA1), G1 (ABCG1) and G4 (ABCG4) (Chen et al., 2013). Subsequently, cholesterol transported by such particles, that resemble plasma HDL in composition and size, is finally incorporated into neurons by the particles binding to specific receptors, such as the LDL receptor (LDLr), the LDL receptor-related protein 1 (LRP1), the VLDL receptor (VLDLr) and the ApoE receptor 2 (ApoEr2) (Bu, 2009). Concerning the latter two, PCSK9 increases their degradation (Poirier et al., 2008; Canuel et al., 2013), implying PCSK9 in cerebral cholesterol homeostasis. This hypothesis is strengthened by *in vivo* findings showing that LDLr expression is reduced by PCSK9 during brain development and after transient ischemic stroke (Rousselet et al., 2011). It is therefore conceivable that the degrading activity of PCSK9 on lipoprotein receptors listed above may translate in a reduced cholesterol uptake by neurons, with potential deleterious consequences (Koudinov and Koudinova, 2005). However, not all data are consistent with this hypothesis. Liu et al. (2010) found that PCSK9 did not affect the expression of LDLR, VLDLR and apoEr2 in the mouse brain (Liu et al., 2010). These discrepancies highlight the need for further studies to dissect out the involvement of PCSK9 on brain cholesterol homeostasis.

## Pcsk9 and Alzheimer’S Disease Pathogenesis

Alterations of CNS cholesterol homeostasis are associated with various neurodegenerative disorders, including AD (Sato and Morishita, 2015; Arenas et al., 2017). Genomic-wide association (GWAS) studies have identified several loci involved in lipid metabolism among AD susceptible genes (Lambert et al., 2013; Dong et al., 2017). A striking example of this association is the ε4 allele of the APOE gene encoding ApoE, the main apolipoprotein mediating the transport of cholesterol in the CNS (Mahoney-Sanchez et al., 2016). The E4 isoform is undoubtedly one of the most predictive factors for AD onset (Liu et al., 2013). However, recent studies have identified other genes involved in lipid metabolism, such as BIN1, CLU, PICALM, ABCA7, ABCA1, ABCG1 and SORL1 (Dong et al., 2017; Picard et al., 2018). From a molecular point of view, the cerebral cholesterol accumulates in lipid rafts, membrane microdomains where the processing of the amyloid precursor protein (APP; Picard et al., 2018) occurs, leading to deposition of insoluble fragments of Aβ in brain parenchyma. At this regards, it has been found that cholesterol promotes amyloidogenesis by providing structural stability to membrane-adjacent lipid rafts (Vetrivel and Thinakaran, 2010). Consequently, modulation of cholesterol content in lipid rafts is able to affect deposition of Aβ.

The few and controversial data on PCSK9 and AD are summarized in Table 1. Concerning neuronal apoptosis, a pro-apoptotic activity of PCSK9 may occur through the upregulation of caspases or the reduction of the ApoEr2 levels (Wu et al., 2014). In APOE^(−/−)^ mice fed with a high-fat diet, the hippocampal neuronal apoptosis was associated with an increase of PCSK9 expression (Zhao et al., 2017). Consistently, silencing of PCSK9 attenuates the neuronal apoptosis induced by cerebral ischemia reducing brain damage in mice (Wang et al., 2018). Conversely, a preventive action of PCSK9 on neuronal apoptosis may occur through the decrease in Aβ generation (Wu et al., 2014). In addition, the direct effect of PCSK9 on Aβ processing is still unresolved. In its absence, mice show increased expression of the β-site amyloid precursor protein-cleaving enzyme 1 (BACE1), the protease producing toxic Aβ that accumulates in neuritic plaques of AD brains. This effect translates in an increased total Aβ brain deposition: PCSK9 overexpression in mice reduced BACE1 levels (Jonas et al., 2008). On the other hand, in brain-damaged rats, the administration of a small molecule inhibiting PCSK9 prevented dendritic spine loss by attenuating the aggregation of Aβ and neuroinflammation (Apaijai et al., 2019). No evidence that PCSK9 regulates BACE1 levels or APP processing in the brain of mice has been reported by other authors (Liu et al., 2010; Fu et al., 2017). To the best of our knowledge, no data are available on the potential influence of PCSK9 on tau phosphorylation, another peculiar hallmark of AD pathogenesis.

**Table 1:** Summary of studies investigating the involvement of PCSK9 in AD pathogenesis.

Study	Experimental model	Involvement of PCSK9 in AD	Effect on cholesterol metabolism and inflammation
Wu et al. (2014)	Mice model	PCSK9 promotes neuronal apoptosis	Reduced apoEr2
Wu et al. (2014)	Mice model	PCSK9 prevents neuronal apoptosis and decreases Aβ generation	
Zhao et al. (2017)	Mice model	Hyperlipidaemia induces neuronal apoptosis by increasing PCSK9 and BACE1 expression	Increased lipid accumulation in the hippocampus
Wang et al. (2018)	Mice model	Inhibition of PCSK9 attenuates the neuronal apoptosis	Reduced ApoEr2 in hippocampus and cortex
Jonas et al. (2008)	Cellular models	Absence of PCSK9 induces Aβ production while its overexpression reduces BACE1 levels	Reduced LDLr
Apaijai et al. (2019)	Rats	PCSK9 inhibitor administration attenuates Aβ aggregation	Reduced number of CD11b^+^/CD45^high^ microglia
Liu et al. (2010)	Mice model	PCSK9 does not regulate BACE1 levels or APP processing in the brain	No effect on LDLr, VLDLr and apoEr2
Fu et al. (2017)	Cellular and mice models	PCSK9 does not regulates APP processing in brain	No effect on LDLr
Reynolds et al. (2010) Shibata et al. (2005) Benn et al. (2017) Mefford et al. (2018) Paquette et al. (2018)	Human (genetic studies)	PCSK9 gene variants are not associated to the risk ratio for AD	
Zimetti et al. (2017)	Human	PCSK9 levels are increased in CSF of AD patients	Correlation between PCSK9 and apoE4. PCSK9 levels higher in APOE ε4 carriers
Courtemanche et al. (2018)	Human	PCSK9 levels are increased in CSF of AD and non-AD neurodegenerative patients	CSF PCSK9 is positively correlated with AD biomarkers

*Abbreviations: AD, Alzheimer’s Disease; BACE1, β-site amyloid precursor protein-cleaving enzyme 1; Aβ, Amyloidβ; CSF, cerebrospinal fluid; apoE, apolipoprotein E; LDLr, low density lipoprotein receptor; VLDLr, very low density lipoprotein receptor; apoEr2; CHO, Chinese hamster ovary; APP, amyloid precursor protein*.

The impact of PCSK9 on neurocognitive performances in pre-clinical models has been indirectly suggested by the observation that deletion of the LRP1, which is sensitive to the degrading action of PCSK9 (Canuel et al., 2013), leads to a reduced Aβ clearance and to cognitive deficits in mice (Storck et al., 2016). Consistently, mice lacking the LDLr show signs of impaired learning and memory (Mulder et al., 2004), reduced hippocampal cell proliferation and synapses formation (Mulder et al., 2007).

### Lipoprotein Receptors Target of PCSK9 and Their Role in Alzheimer’s Disease

**Figure 1:**
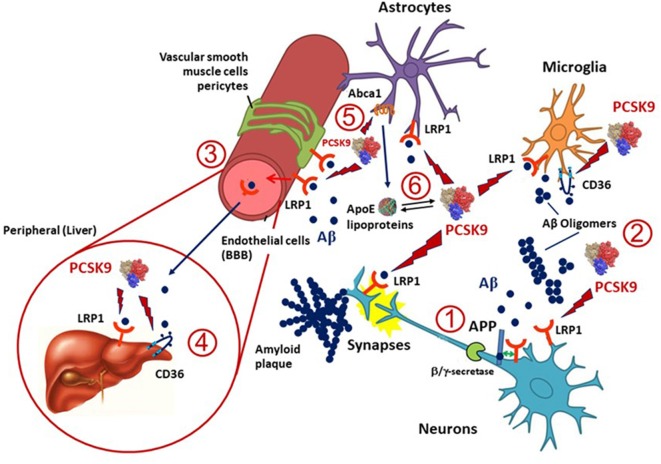
Potential implication of proprotein convertase subtilisin/kexin type 9 (PCSK9) in amyloid β (Aβ) clearance in Alzheimer’s disease (AD). LDL receptor-related protein 1 (LRP1), expressed in microglia, neurons, astrocytes and pericytes, and CD36, mainly present in microglia, are the two main lipoprotein receptors involved in Aβ clearance and are potentially targeted by PCSK9. (1) LRP1 may also influence the production of Aβ from amyloid precursor protein (APP) in neurons through a direct protein-protein interaction or competition with the α/β-secretase cleavage of APP. (2) Once Aβ is released into the extracellular space in the brain can form amyloid plaques or oligomers and LRP1 or CD36 can mediate its cellular uptake by neurons, microglia, astrocytes, vascular smooth muscle cells, pericytes and endothelial cells. (3) A portion of Aβ may be transported through LRP1 at the blood-brain barrier (BBB) and reversed into the blood, thus PCSK9 may also interfere with this process. (4) Both LRP1 and CD36 expressed in the liver might also help the clearance of Aβ from the blood, and PCSK9 may affect this pathway by reducing their expression levels in hepatocytes. (5) Apolipoprotein E (ApoE), which is mainly produced and secreted from astrocytes in the brain, is lipidated by ATP binding cassette transporters A1 (ABCA1) to supply cholesterol/lipids to neurons and other cells through LRP1 and CD36. PCSK9 has been shown to downregulate the expression of ABCA1, thus opening to a possible modulation of the release of ApoE containing lipoproteins and thus LRP1- or CD36-mediated Aβ metabolism. Indeed, ApoE isoforms may affect LRP1-mediated Aβ metabolism by directly interacting with Aβ or competing with Aβ for receptor binding. (6) ApoE lipoprotein may also interact with PCSK9 hence influencing its action on LRP1 and CD36.

Besides APOE, several cholesterol homeostasis related genes have been investigated for their association with AD (Wollmer, 2010) and some of them are targeted by PCSK9 (Figure 1). LRP1 is an ApoE receptor, and both positive and negative results on its genetic association with AD have been reported (Kang et al., 1997). LRP1 expression is reduced in total brain and brain capillaries with increasing age and even more reduced in AD (Kang et al., 2000; Shibata et al., 2000; Silverberg et al., 2010). Highly validated genetic risk factors for AD, like the APOE allele, may be linked to a reduced clearance of Aβ *via* LRP1 (Bell et al., 2007; Deane et al., 2008). LRP1 mediates Aβ clearance from the brain into the circulation at the BBB (Deane et al., 2009) and brain endothelial-specific LRP1 deletion elevates soluble brain Aβ, leading to aggravated spatial learning and memory deficits (Storck et al., 2016). Thus, LRP1 plays a pivotal role in the metabolism of the ApoE-Aβ complex and ApoE may compete with Aβ for the interaction with LRP1, resulting in impaired Aβ clearance (Verghese et al., 2013). LRP1 is also involved in the hepatic clearance of Aβ (Sagare et al., 2007). Because LRP1 is expressed in different cell types, including neurons, astrocytes and vascular cells in the brain, its levels may be altered differently in AD (Donahue et al., 2006; Ruzali et al., 2012). Indeed, LRP1 levels are decreased in neurons but increased in vascular cells or astrocytes that are proximate to amyloid plaques in AD brains (Arélin et al., 2002; Donahue et al., 2006; Ruzali et al., 2012). PCSK9 may induce the degradation of LRP1 in different cell types, including hepatocytes (Canuel et al., 2013) and vascular cells (Ferri et al., 2012; 2016b). Since PCSK9 is expressed in neurons and in vascular cells, it may influence the LRP1 levels in these cell types (Poirier et al., 2009). Thus, the observed increased PCSK9 cerebrospinal fluid (CSF) concentrations in AD (Zimetti et al., 2017) may determine a higher turnover of LRP1 on different cell types, thus affecting the Aβ elimination *via* the BBB.

The LDLr is a major ApoE receptor in the brain and genetic studies aiming at identifying its link to AD are controversial, although one large study found an association in men but not in women (Lendon et al., 1997; Zou et al., 2008). In a mouse model of AD, LDLr demonstrated a beneficial effect *via* enhancement of Aβ clearance (Kim et al., 2009), suggesting its promising association with the risk for AD and consequently the involvement of PCSK9.

VLDLr can be involved in the clearance of ApoE-Aβ complexes (Helbecque and Amouyel, 2000). However, a meta-analysis of genetic studies conducted on the VLDLr gene polymorphic triplet (CGG) repeat in the 5-UTR showed contradictory results, being a protective factor for AD in Caucasians and as a risk factor in Asian people (Llorca et al., 2008). In addition, genetic association with AD was described in one study but not confirmed in two replication studies (Taguchi et al., 2005). Thus, the direct association of VLDLr and AD still needs to be proven.

The scavenger receptor CD36 is involved in fibrillar Aβ-mediated microglial activation and consecutive activation of an innate immune response (Coraci et al., 2002; Moore et al., 2002; Bamberger et al., 2003). PCSK9 leads to increased CD36 expression in macrophages and microglial-like cells (Ding et al., 2018), suggesting that PCSK9 may regulate both the CD36-mediated clearance of Aβ and the innate host response to Aβ and oxidized-LDL (oxLDL) in brain cells. Indeed, CD36 acts as a co-receptor for the toll-like receptors (TLRs) heterodimerization, an essential step for the initiation of the inflammatory signals and microglia-dependent neurodegeneration (Stewart et al., 2010). Consistently, PCSK9 elicits a proinflammatory effect on macrophages (Ricci et al., 2018) and the administration of a PCSK9 inhibitor leads to a neuroinflammation attenuation in mice models (Apaijai et al., 2019).

Finally, our group reported that human recombinant PCSK9 inhibits the ABCA1-mediated cholesterol efflux in macrophages (Adorni et al., 2017). In this regard, data on the influence of ABCA1 in AD are conflicting as well. For example, carriers of the R219K SNP in the ABCA1 gene shown 33% lower total cholesterol in CSF compared to non-carriers. This allele is also associated with a delay of disease onset by 1.7 years on average (Wollmer et al., 2003). This suggests that a genetic variability of ABCA1 influences the development of AD, possibly by interfering with CNS cholesterol homeostasis. Nevertheless, an association of the same ABCA1 variant R219K with increased AD risk has been described (Rodríguez-Rodríguez et al., 2007). Similarly, additional evidence reported the association between genetic variants of ABCA1 gene with either reduced or increased AD risk (Katzov et al., 2004; Nordestgaard et al., 2015; Beecham et al., 2018). Specifically, a genetic study involving more than 90,000 subjects evidenced a significant association between ABCA1 loss-of-function mutation and 41% increased risk of AD (Nordestgaard et al., 2015). Nevertheless, also for ABCA1, several negative studies have been published, such as a meta-analysis where no association has been found between R219K, I883M and R1587K polymorphisms and risk of AD (Jiang et al., 2012). The involvement of ABCA1 in AD has been investigated in ABCA1 knock-out mice cross-bred with an amyloid pathology of AD. The absence of ABCA1 determined a higher amyloid load in the brains (Koldamova et al., 2005; Wahrle et al., 2005, 2008), although other similar experiments failed to show an effect of ABCA1 on amyloid pathology (Hirsch-Reinshagen et al., 2005, 2007). Since ABCA1 regulates ApoE levels and the transfer of cholesterol from the glial to the neuronal compartment (Wahrle et al., 2004), its role on AD may involve PCSK9, which affects ABCA1 expression (Adorni et al., 2017). Consistent with this hypothesis, a strong decrease in ApoE levels in both the cortex and CSF, together with an impairment of its lipidation, has been described in ABCA1 knock-out mice (Wahrle et al., 2004). Interestingly, CSF samples extracted from AD patients have lower *ex-vivo* capacity to promote ABCA1-mediated cholesterol efflux compared to controls (Yassine et al., 2016). Finally, a further level of complexity could be the possible binding between PCSK9 and apoE-containing lipoproteins as previously described for LDL (Tavori et al., 2013), Lipoprotein (a) (Tavori et al., 2016) and HDL (Ferri et al., 2016a; Ruscica et al., 2018).

### PCSK9 and Alzheimer’s Disease in Humans

The genetic studies conducted so far in humans (Table 1) are not conclusive on the impact of PCSK9 mutations on AD. Although Wollmer (2010) first identified PCSK9 among the cholesterol-related genes that have been matched with AD genes listed in the AlzGene database, no association was found between PCSK9 polymorphism and the risk of AD onset, neither in a Japanese nor in a Swedish cohort study (Shibata et al., 2005; Reynolds et al., 2010). Consistently, in a recent Mendelian randomization analysis, PCSK9 loss-of-function mutations were not associated to a rise in the risk of AD [Hazard Ratio (HR) = 0.50; *p* = 0.37; Benn et al., 2017].

To a negative conclusion came also the results of genetic studies among African American REGARDS (Reasons for Geographic and Racial Differences in Stroke) participants with and without the PCSK9 loss-of-function variants C697X or Y142X. The presence of these variants did not affect the primary endpoint of the study, i.e., the neurocognitive performance (Mefford et al., 2018). In another study conducted in French Canadian subjects, carriers of the PCSK9 loss of function mutations, R46L and InsLEU, did not differ from non-carriers as either AD prevalence or age of disease onset (Paquette et al., 2018).

In humans, PCSK9 has been detected in CSF even though at a much lower concentration compared to plasma (Chen et al., 2014). CSF PCSK9 concentrations appeared to be constant throughout the day, thus not undergoing the typical diurnal pattern of plasma PCSK9 (Persson et al., 2010) and suggesting a differential mechanism of PCSK9 regulation in the peripheral and central body compartments (Chen et al., 2014).

In a previous work, we demonstrated increased levels of PCSK9 in the CSF of AD patients with the highest levels in APOE ε4 carriers (Zimetti et al., 2017). These data suggest an involvement of PCSK9 in the disease and a pathophysiological link with APOE4 that deserves further investigations. Our observation has been confirmed by Courtemanche et al. (2018), however, they show a trend for increased CSF PCSK9 levels also in non-AD neurodegenerative disease, confirming a link to the neurodegenerative process but not specifically to AD.

### PCSK9 Inhibitors and Neurocognitive Disorders

Plasma cholesterol under the PCSK9 inhibitor (alirocumab, evolocumab or bococizumab) treatment reached very low levels (mean level 30 mg/dL in the FOURIER trial and 25 mg/dL in ODYSSEY LONG-TERM and SPIRE trials) (Robinson et al., 2015; Ridker et al., 2017; Sabatine et al., 2017). These observations raised some concerns about the potential side effects related to the clinical use of PCSK9 inhibitors. In particular, some clinical trials highlighted a potential association between treatment with PCSK9 monoclonal antibodies and cognitive adverse events (Robinson et al., 2015; Sabatine et al., 2015). However, such disorders were often self-reported and occurred in very few patients with pre-existing medical conditions or other confounders, as emerged from the results of an analysis made by the FDA (Food and Drug Administration Briefing Document, 2015a,b). In order to better clarify this issue, a recent study prospectively and objectively evaluated the effect of the PCSK9 inhibitor evolocumab on cognitive functions (Giugliano et al., 2017a). In this study, a total of 1,204 subjects with mean age of 65 years, that did not present neurological disorders on treatment with evolocumab or placebo, were followed for 1.6 years without evidencing any association with adverse cognitive effects (Giugliano et al., 2017b). This result was further confirmed by a recent meta-analysis (Bajaj et al., 2018; Harvey et al., 2018). However, considering the short follow-up period of the EBBINGHAUS, a 5-year extension of the FOURIER trial will provide further findings on neurocognitive functions^1^.

The lack of an evident effect by PCSK9 inhibitors on cognitive functions is very likely explained by the BBB presence, with the consequence that high or low levels of cholesterol in the circulation are not likely to have direct effects on lipid level in the brain (Olsson et al., 2017). With this respect, in the genetic study with carriers of PCSK9 loss-of-function variants, lifelong exposure to low levels of LDL-cholesterol (LDL-C) was not indeed associated with neurocognitive effects (Mefford et al., 2018; Paquette et al., 2018).

Moreover, BBB limits the access of both PCSK9 (Rousselet et al., 2011) and more so of monoclonal antibodies, such as alirocumab or evolocumab to the CNS. Under the conditions where the integrity of BBB is intact, the presence of tight junctions prevents the transcellular route for diffusion of antibodies across the capillary. Therefore, in general, the antibodies penetration into the brain has been estimated to be about 0.1%, both in humans and animals (Tabrizi et al., 2010). In some pathological conditions, such as diabetes, the BBB might be compromised (Rom et al., 2019). However, some indications ruling out the possibility that the antibodies cross the BBB in such conditions, come from the EBBINGHAUS study, which also involves diabetic subjects (37.2%), and in which no variation of cognitive functions was observed (Giugliano et al., 2017b).

Thus, it would be of great interest to evaluate the effect of small molecules PCSK9 inhibitors capable of crossing the BBB.

## Conclusion

Although several extrahepatic effects of PCSK9 beyond LDL-C (Stoekenbroek et al., 2018) have been identified and well characterized, its role in the brain and the potential involvement in CNS diseases is still under investigation. In this regard, pathophysiological studies on pre-clinical models of AD led to controversial results, leaving open the question of the potential implication of PCSK9 in the disease pathogenesis. Furthermore, the few genetic studies available focused only on PCSK9 genetic variants leading to loss-of-function mutations and are not supportive of an association between PCSK9 and AD risk (Mefford et al., 2018; Paquette et al., 2018). Notably, in all of the studies, only plasma PCSK9 concentrations have been evaluated, while PCSK9 cannot cross the BBB and its regulation may be different in the central and periphery body compartments. Based on the observation made by ourselves and others regarding increased PCSK9 levels in the CSF of AD patients and considering that PCSK9 may interfere with CNS cholesterol transport by degrading the neuronal ApoE-receptors responsible for astrocyte-derived cholesterol uptake, it is conceivable to hypothesize a PCSK9-induced impairment of cholesterol supply to neurons occurring in AD. The consequences of this cholesterol-depletion would include a loss of neuronal physiological functions and ultimately neurodegeneration. In addition, PCSK9 may contribute to exacerbate neuroinflammation, possibly acting on the receptors CD36 and TLR4 (Stewart et al., 2010). These hypotheses, that are being tested by our research group, may set the basis for testing new pharmacological approaches, including existing small molecules potentially able to cross the BBB by either diffusion or transporter-mediated processes, differing from the anti- PCSK9 antibodies. These molecules, by restoring the physiological brain cholesterol transport from astrocytes to neurons in CNS and by attenuating the neuroinflammation through CD36-TRL4 pathway inhibition, might clear the path for a potential future innovative AD therapy.

## Author Contributions

FZ and MA wrote the first draft of the review and prepared the table. NF, MR and FB wrote sections of the review article. NF made the figure. All authors critically revised the text and all approved the submitted version.

## Conflict of Interest Statement

FB received a financial grant from Amgen (Amgen’s PSCK9 Competitive Grant Program 2018) with a project entitled “EXplorIng the paThophysological role of PCSK9 in Alzheimer’s Disease focus on inflammation and lipid metabolism (EXIT-AD)”.

The remaining authors declare that the research was conducted in the absence of any commercial or financial relationships that could be construed as a potential conflict of interest.

## Footnotes

^1^https://ClinicalTrials.gov/show/NCT02867813
